# Comparative Analysis, Characterization and Evolutionary Study of Dirigent Gene Family in Cucurbitaceae and Expression of Novel Dirigent Peptide against Powdery Mildew Stress

**DOI:** 10.3390/genes12030326

**Published:** 2021-02-24

**Authors:** Vivek Yadav, Zhongyuan Wang, Xiaozhen Yang, Chunhua Wei, Xuan Changqing, Xian Zhang

**Affiliations:** 1 State Key Laboratory of Crop Stress Biology in Arid Areas, College of Horticulture, Northwest A & F University, Yangling 712100, China; vivekyadav@nwafu.edu.cn (V.Y.); zydx@nwafu.edu.cn (Z.W.); xiaozhen.yang@nwsuaf.edu.cn (X.Y.); xjwend020405@nwafu.edu.cn (C.W.); xuanchangqing@nwafu.edu.cn (X.C.); 2 Xi’an Agriculture Technology, Extension Center, Xi’an 710000, China; 3 State Key Laboratory of Vegetable Germplasm Innovation, Tianjin 300384, China

**Keywords:** dirigent domain, powdery mildew, gene family, expression pattern, Cucurbitaceae, watermelon

## Abstract

Dirigent (DIR) proteins are induced under various stress conditions and involved in sterio- and regio-selective coupling of monolignol. A striking lack of information about dirigent genes in cucurbitaceae plants underscores the importance of functional characterization. In this study, 112 DIR genes were identified in six species, and 61 genes from major cultivated species were analyzed. DIRs were analyzed using various bioinformatics tools and complemented by expression profiling. Phylogenetic analysis segregated the putative DIRs into six distinctively known subgroups. Chromosomal mapping revealed uneven distribution of genes, whereas synteny analysis exhibited that duplication events occurred during gene evolution. Gene structure analysis suggested the gain of introns during gene diversification. Gene ontology (GO) enrichment analysis indicates the participation of proteins in lignification and pathogen resistance activities. We also determined their organ-specific expression levels in three species revealing preferential expression in root and leaves. Furthermore, the number of CmDIR (*CmDIR1*, *6*, *7* and *12*) and ClDIR (*ClDIR2*, *5*, *8*, *9* and *17*) genes exhibited higher expression in resistant cultivars after powdery mildew (PM) inoculation. In summary, based on the expression and in-silico analysis, we propose a role of DIRs in disease resistance mechanisms.

## 1. Introduction

Dirigent protein models suggest that lignin oligomers biosynthesis is highly controlled by dirigent (DIR) genes. The Dirigent proteins help in the formation of chemical bonds during the polymerization process of monolignol [1,2,3]. These proteins are induced under various stress conditions and directly involved in the sterio-selective coupling of monolignol to produce basic monolignol units and contribute to stereoselective biosynthesis for example (+) or (−) pinoresinol from coniferyl alcohol [4]. ‘Dirigent’ term is derived from the Latin term “dirigere” which means ‘to guide’. The first discovered DIR protein was found to be involved in mediating free radical coupling of monolignol phenols for the formation of (+) pinoresional, dimer known as lignans, in which monomers are linked by C8 central carbon atom of the propyl side chains [5,6,7]. A wide distribution of DIR proteins was found in all vascular plants, including ferns, gymnosperms and angiosperms [8].

Lignification is typically associated with the structural resistance to pathogens and it plays a crucial role against pathogens attack by inhibiting microbe-derived degradative enzymes such as cellulases, glucosidases, laccases, and polygalacturonases [9,10,11]. Lignins are cell wall components, whereas lignans are low molecular, water soluble polyphenolic molecules that provide basal defense during plant–pathogen interaction [8]. Several studies have described that lignin positively contributes towards plant defense. For example, essential contribution of lignin was demonstrated in *Medicago sativa*, and it was observed that selective down regulation of lignin biosynthesis pathway genes resulted in systematic expression of disease resistance genes [12]. Lignans have antioxidant, antimicrobial and antifeedant properties [13]. The involvement of DIRs in the biosynthesis of lignin units and lignans has attracted the scientific community to uncover the functions of these proteins in various stress tolerance mechanisms.

Nevertheless, many studies demonstrated the role of DIRs in plant–pathogen interaction. For instance, the induction in DIR-like genes was observed along with modification in cell-wall after the invasion of moss *Physcomitrella patens* with *Colletotrichum gleosporioides* [14]. A recent study showed that the four DIR proteins were induced by *Pectobacterium carotovorum* elicitors-treated moss [15]. An increase in activity of DIR-4 was observed upon infection by *C. gleoosporioides* and *Botrytis cinerea* [14,16]. Similarly, microarray-based gene expression studies showed differential expression in DIR-like genes and induction in *BrDIR12* gene after infection of *Fusarium oxysporum* [17]. In rice, *OsJAC1* has been studied and revealed to contain a dirigent domain. The overexpression of *OsJAC1* resulted in broad spectrum resistance against multiple pathogen attack [18]. Similarly, an important study about the overexpression of ortholog (*TaJAC1*) gene of wheat resulted in enhanced powdery mildew (PM) resistance in barley [18]. Moreover, in some studies it is suggested that DIRs could contribute significantly against fungus attack in plants. For example, when flax cell was exposed to *F. oxysporum*, an increase in expression of *LuDIR24* and 36 was observed [19]. Evidently, higher expression in DIRs leads to pinoresinol accumulation after pathogen invasion to plants [19]. 

The plant basal defense system is a result from the activation of multiple defense pathways, which involves the induction of multiple genes, including DIR genes. Dirigent is found to be a part of an important disease resistance responsive gene (DRRG) that plays a key role in different stress conditions [20]. These novel peptides do not show a significant level of resemblance to any other protein of known function. The role of dirigent proteins to mediate stereoselective phenol coupling is well documented and understood. However, their role in plant defense interaction is still poorly understood and no detailed information is available for its function in cucurbitaceae.

Cucurbit crop growing area in the field and protected cultivation is almost two times higher than the growing area of tomatoes. Cucurbits constitute the largest group of tropical vegetables comprising popular and economically important species of vegetable crops, including cucumber (*Cucumis sativus*), muskmelon (*Cucumis melo*), watermelon (*Citrullus lanatus*), different gourds and squashes. Cucurbits species are easily infected with powdery mildew (PM) fungus. Powdery mildew is a devastating fungal disease that can affect the crop quality and production of all major cucurbit crops. PM is a major foliar pathogen and it can easily infect stems, petioles, cotyledons, hypocotyl, and fruits [21]. Infection in the initial stages can cause poor growth and reduced vigor [22,23]. Powdery mildew is a constant threat to almost all the growing areas around the world and it can be observed throughout the year in many growing regions [23]. 

The functional analysis of DIR genes in plant defense processes could provide a foundation approach to improving defense response against various biotic stresses. However, little information is available in public domains about cucurbit DIRs and their possible involvement in defense mechanisms against powdery mildew. To this end, we carried out the systematic genome wide analysis of DIRs and their expression divergence pattern. Along with the identification of family members in six important species, we analyzed gene structure, chromosome mapping, phylogenetic evolution, conserved motifs, gene structure, and gene duplications in three most important species. The expressions of DIRs in different tissues of watermelon, melon and cucumber were investigated. Furthermore, comparison of expression levels among contrasting cultivars of melon and watermelon infected with PM were also presented. We attempted to portray a complete picture to provide a comprehensive understanding of DIRs and explore their possible functions in plant–pathogen interaction. 

## 2. Materials and Methods 

### 2.1. Plant Materials and Disease Infection 

ZXG1996 (PM- susceptible genotype) and M16 (PM-resistant genotype) were obtained from the cucurbit research laboratory, College of Horticulture, Northwest A & F University [24]. Seeds of the selected germplasm were germinated in a germination chamber after soaking it for one day in the dark. Subsequently, the germinated seeds were transferred to a 50-cell tray. Seedlings with two true leaves were transferred to plastic pots (10 × 7 × 8.5 cm) filled with a mixture of 2:1 (v/v) autoclaved sand and commercial peat-based compost (Shaanxi Yufeng Seed Industry Co., Ltd., Yangling, China). The seedlings were kept in a growth chamber under 70–90% relative humidity, a photoperiod of 16 h light/8 h dark, and air temperatures of 29/19 °C (day/night). PM (*Podosphaera xanthii*) inoculation experiment was conducted using the ‘2F’ race of cucurbit PM, maintained on the watermelon plants in the greenhouse. The seedlings were sprayed with a conidial suspension (10^5^ conidia/mL with 0.02% Tween 20) as described in previous research [25,26,27]. Finally, the infected leaves samples were collected in three replications at 0, 24, 48, 72, 96, and 120 h post-inoculation (hpi). Harvested samples were frozen immediately in liquid nitrogen and stored for RNA extraction at −80 °C. 

### 2.2. Sequences Assembly of DIRs in Cucurbitaceae

The downloaded reference genome assembly and protein sequence from the cucurbit genome database (http://cucurbitgenomics.org/) were used for primary identification of all members of DIRs in major cucurbitaceae species. Hidden Markov Model (HMM) of the dirigent domain (PF03018) was downloaded from the Pfam protein family database (http://pfam.sanger.ac.uk/) [28] and used to identify the putative candidates using the HMMER search program with E-value threshold of 0.01 (HMMER 3.0; http://hmmer.janelia.org/) [29,30]. The NCBI CDD search (https://www.ncbi.nlm.nih.gov/Structure/bwrpsb/bwrpsb.cgi) [31], Pfam [28], and SMART (http://smart.embl-heidelberg.de/) were used to further confirm the conserved DIRs domain. Finally, all the candidates were selected after removing the redundant sequences [32].

### 2.3. Physical Location of DIR Genes on the Chromosome and Synteny Analysis

The chromosome locations of DIR genes in different cucurbit species were obtained from cucurbit genome database (http://cucurbitgenomics.org/), and the physical location of each gene was plotted using Mapgene2Chromosome V2.1 (http://mg2c.iask.in/mg2c_v2.0/) on a specific chromosome of *C. lanatus*, *C. melo* and *C. sativa*. Annotation of the putative genes was assigned based on their chromosome position and number [33]. Related synteny blocks and duplicated gene pairs in Arabidopsis, cucumber, melon, and watermelon were obtained using TBtools software. For collinearity analysis, the relationships between major species, including *C. melo*, *C. lanatus*, *C. sativa*, and *Arabidopsis thaliana* were verified and visualized by Circos tool (http://circos.ca/) [32].

### 2.4. Predicted Protein Structure, Property Analysis and Subcellular Localization

The protein properties like molecular weight (MW), CDS length (bp), isoelectric point (*p*I), number of amino acids, instability index, aliphatic index, major amino acid and GRAVY were obtained from Protparam online tool (https://web.expasy.org/protparam/) [34]. WoLF PSORT II (https://www.genscript.com/wolf-psort.html) online server was used to predict the subcellular locations. To ensure the reliability, the subcellular localization of identified DIR proteins was checked by Cell-Ploc2.0 (http://www.csbio.sjtu.edu.cn/bioinf/Cell-PLoc-2/). 

### 2.5. Phylogenetic Analysis, Motif, and Gene Structures 

The phylogenetic tree was constructed using the DIR protein sequences of several crops including 22 watermelon, 22 melon, 17 cucumber, 25 Arabidopsis, rice and protein sequences from various identified plant species (Table S2). The MEGA-X software [35] was used with the following parameters to construct the phylogenetic tree; neighbor-joining (NJ) method, 1000 bootstrap replicates, ‘*p*-distance,’ ‘Complete Deletion,’ and gap setting [36]. The phylogenetic tree was displayed using iTOL (https://itol.embl.de/) online tool. The CDS sequences of DIRs were obtained from the genome database, and motif prediction was undertaken with online MEME server V 4.12.0 (http://meme-suite.org/tools/meme) tool with default parameters and the maximum number of motifs was set to 10. The gene structure of each DIR in three species of cucurbitaceae was illustrated by Gene Structure Display Server (GSDS) (http://gsds.gao-lab.org) [37]. 

### 2.6. Gene Ontology and Cis-Element in Promoter Region

GO analysis was done for the functional annotation of DIR protein sequences using Blast2GO program Ver. 2.7.1 (http://www.blast2go.com). To depict the functional involvement of DIR genes, GO terms associated with each group of the obtained hit were analyzed based on their cellular, molecular and biological processes and presented in the form of a pie-chart. Next, the potential *cis*-regulatory elements were obtained by scanning 2.0 kb upstream sequence from the transcription start site (start codon ATG) on PlantCARE program (http://bioinformatics.psb.ugent.be/webtools/plantcare/html/).

### 2.7. Quantitative Real-Time PCR

The inoculated leaf samples of watermelon germplasm ZXG-1996 (susceptible) and M16 (resistant) were collected at 0, 12, 24, 48, 72, 96, and 120 h post-inoculation. The samples were harvested from multiple plants and pooled together as a single biological replicate at each inoculation stage. Three biological replicates were used for all the time periods. The samples were immediately frozen in liquid nitrogen and stored at −80 °C. The total RNA of the leaves was extracted using the RNA Simple Total RNA Kit (TIANGEN, China) following the manufacturer’s instructions. cDNA synthesis was done with FastKing RT Kit with gDNase (TIANGEN, China). The gene sequences used in this study were retrieved from the Cucurbit Genomics Database (http://cucurbitgenomics.org/). The qRT-PCR gene specific primers were designed by using Primer3Plus (https://primer3plus.com/cgibin/dev/primer3plus.cgi) (Table S3). The reactions were performed in a 20 µL volume consisting of 0.8 µL of each primer (10 µM), 10.0 µL SYBR Green Premix and 1.0 µL cDNA template (80 ng/µL), which was diluted 20 times with ddH_2_O. The qRT-PCR conditions consisted of predenaturing at 95 °C for 5 min, followed by 40 cycles of 95 °C for 10 s and 60 °C for 30 s. The relative gene expression of each gene was determined using 2^−ΔΔ*C*T^ method. *Actin* used as an internal control [32,33,38]. 

### 2.8. Expression Analysis of DIR Genes in Various Tissue under PM Stress

The watermelon plants were cultivated on the experimental farm, and the different plant tissues (root, shoot, branch. tendril, male flower, female flower, and leaf) were collected for tissue-specific expression. Moreover, RPKM (reads per kilobase of exon per million mapped reads) values of CmDIR and CsDIR genes in various vegetative and reproductive tissues of *C. melo* and *C. sativa* were obtained from transcriptome database (http://cucurbitgenomics.org/). Furthermore, the expression pattern of the DIR genes was performed in contrasting cultivars (resistant-M16 and susceptible-ZXG1996) of *C. lanatus*, and were compared under PM infection. Similarly, the transcriptome data for the melon cultivars Topmark (sensitive) and MR-1 (resistant) were obtained from the cucurbit genome database, and a heat map was constructed after normalization.

### 2.9. Statistical Analysis

SPSS version 25 (SPSS Inc., Chicago, IL, USA) statistics program was used to analyze multiple data comparison using analysis of variance (ANOVA) with significance level *p* < 0.05, and were expressed as the mean ± SE.

## 3. Results

### 3.1. Genome-Wide Analysis Reveals Diversification of DIR in Cucurbitaceae 

DIR gene family members were identified by using dirigent protein conserved domain (PF03018) profile as a query for Hidden Markov Model (HMM) to identify the dirigent family members. The search was carried out with HMMER suit version 3.0. The sequences were considered positive when the score was greater than or equal to the reported gather score. The redundant, overlapped, incomplete, and repeated sequences were rejected. Finally, the sequences were manually reaffirmed using Interproscan and SMART blast to reconfirm the presence of the dirigent domains in each sequence. In this way, 22, 22, 17, 20, 19, and 23 DIR genes in *C. lanatus*, *C. melo*, *C. sativus*, *C. pepo*, *C. moschata*, *L. siceraria* were retrieved (Table S1). The obtained genes were designated based on the order of the chromosome number and position on the chromosome 

### 3.2. Physiochemical Properties of DIR Proteins and Prediction of Tertiary Structures 

Essential characteristics of 61 DIR proteins from three species of cucurbits are presented in Table 1. In case of ClDIRs, the CDS length ranged from 1191(*ClDIR06*) to 474 (ClDIR01), and the average lengths of the sequences ranged from 300 to 500 bp. A massive difference between the length of amino acid was observed. The length of amino acids ranged from 157 (ClDIR1) to 301 (ClDIR19). The molecular weight of proteins ranged from 17,273.59 to 41,460.80. The isoelectric point values ranged from 4.92 to 10.04. Likewise, in the other two species, the CDS length varied from 1185 to 519 and the amino acid length varied from 394 to 154. Whereas the instability index and aliphatic index in all three species varied from 9.42 to 52.29 and 68.86 to 100.64, respectively. Proteins, including ClDIR03, CmDIR20, and CsDIR05 were stable in nature with lower instability index. Few proteins from each species were hydrophilic in nature, with the Grand Average of hydropathicity (GRAVY) below 0. Our results indicated that the majority of DIR proteins contain signal peptide in their sequence, and few DIR proteins have transmembrane alpha helix. The subcellular localization prediction of each member of ClDIR, CmDIR and CsDIR was performed and it was found that the majority of the DIR proteins of cucurbit species were located in chloroplast followed by extracellular space. Majority of ClDIR proteins were located in extracellular space, whereas large number of melon and cucumber DIR proteins were located in chloroplast with few exceptions (Table 1). ClDIR01, ClDIR7, CmDIR05 proteins of watermelon were predicted to be located in the chloroplast. The prediction of the subcellular location showed that 1(ClDIR19), 2(CmDIR09, 12), and 1(CsDIR01) DIR proteins of watermelon, melon, and cucumber are likely located in the nucleus.

### 3.3. Sequence Alignment and Evolutionary Relationship of DIR Genes

**Figure 1:**
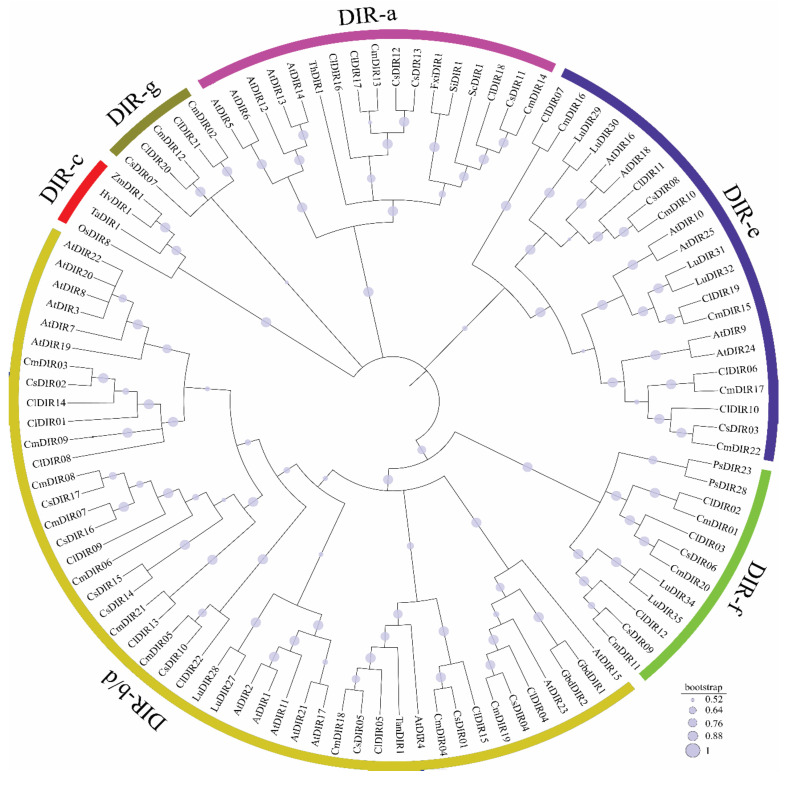
Phylogenetic tree of dirigent proteins identified in different plant species. The evolutionary relationship between different species were inferred using neighbor-joining method by software MEGA-X. The tree was constructed with 1000 bootstrap replications. The evolutionary distances were computed using Poisson correlation method. Six unique groups were identified with high reliability are highlighted in different colors. Plotting was done with online iTOL (https://itol.embl.de/). Dirigent nomenclature is as follows: At—*Arabidopsis thaliana*; Cs—*Cucumis sativus*; Cl—*Citrullus lanatus*; Os—*Oryza sativa*; Cm—*Cucumis melo*; Ta*—Triticum aestivum;* Hv*—Hordeum vulgare;* Zm*—Zea mays;* Lu*—Linum usitatissium;* Sc*—Schisandra chinensis;* Si*—Sesamum indicum;* Fxi*—Forsythia intermedia;* Tan*-Tamarix androssowii*.

To further characterize and identify potential functional relationships between the DIR proteins of cucurbitaceae, all the DIR protein sequences corresponding to 22 watermelon, 22 melon and 17 cucumber were used to construct a phylogenetic tree, together with 47 previously characterized DIR proteins of *A. thaliana* and other selected plant species (Figure 1). The neighbor-joining method with 1000 bootstrap reconstruction and completed deletion gaps/missing data, yielded six known subfamilies. The total of 108 DIR proteins were clearly categorized into five well conserved subgroups (DIR-b/d considered as a single group) and one species-specific group based on sequence relatedness or similarities in functional behavior among subgroups. The analysis revealed that DIR proteins from cucurbits were well distributed in six subfamilies from DIR-a to DIR-g and the majority of the DIR from different species of cucurbits clustered to DIR- b/d subfamily followed by DIR-a and DIR-f classification. The classification system of subfamilies was followed according to the previous classification of *O. sativa* and *A. thaliana* and other orthologs genes of different plant species [3,39].

#### 3.3.1. DIR-a

The DIR-a subfamily contained 5 (AtDIR12, AtDIR5, AtDIR6, AtDIR13, AtDIR14) [3], 3 (ClDIR16, 17, 18), 3 (CsDIR11, 12, 13), and 2 (CmDIR13, 14) DIR proteins from Arabidopsis, watermelon, cucumber, and melon, respectively. Moreover, the DIR-a cluster contained some more proteins such as *Schisandra schinensis* (ScDIR1), *Sesamum indicum* (SiDIR1), *Forsythia intermedia* (FxiDIR1), *Tsuga heterophylla* (ThDIR1) [19]. Based on the classification system of Arabidopsis and other mentioned species, the aforementioned DIR proteins were categorized as DIR-a subfamily and 3, 2, and 2 DIR proteins from watermelon, cucumber, and melon, respectively, were found in this subfamily (Figure 1).

#### 3.3.2. DIR-b/d

As shown in Figure 1, the majority of DIR proteins from all the species falls under this group. This group is sometimes clustered with DIR-d into a single cluster DIR-b/d [19]. Nine (*ClDIR01*, *4*, *5*, *8*, *9*, *13*, *14*, *15*, and *22*) proteins from watermelon, 10 (*CsDIR01*, *2*, *4*, *5*, *10*, and *14–17*) from cucumber and 10 (*CmDIR03-09*, *18*, *19*, and *21*) melons proteins were classified in this large group. However, this group was enclosed in a wider group of highly similar proteins which also include four (*AtDIR1*, *2*, *11*, *17*, and *21*) from DIR-d group. This wide group was classified on the basis of classification done in many other species such as *C. annuum* [29], *O. sativa* [39], *A. thaliana* [3], *L. usitatissium* (LuDIR27, 29*)*, *Tamarix androssowii* (TmaDIR01), and *Gossypium bardadense* (GbdDIR1 and 2) [19]. 

#### 3.3.3. DIR-c

DIR-c is monocot specific family, and no proteins were classified in this subfamily from dicot plant species. However, *L. usitatissium* (LuDIR24) was found to be closely aligned to members of this group, the sequence divergence was reported higher, and it was stated that LuDIR24 is not linked to any group [19]. The previous studies done in rice, maize, barley, and wheat demonstrated the presence of the number of DIR proteins in this subfamily. We included some exclusive genes from these groups to support our classification of cucurbit DIR genes (Figure 1). Notably, no proteins from our study were reported in this monocot specific DIR group.

#### 3.3.4. DIR-e

Six DIR proteins (AtDIR16, 18, 25, 24, 10, and AtDIR9) of Arabidopsis along with two proteins CsDIR03 and 08 from cucumber, five proteins CmDIR17, 22, 15, 16, and 10 from melon and five proteins ClDIR10,11, 6, 17, and 19 from watermelon were clustered in the same group. This classification was based on the dirigent gene classification of *A. thaliana* and *L. usitatissium* (Figure 1) [3,19,20].

#### 3.3.5. DIR-f

Three proteins from melon (CmDIR01, 11 and 20) and three from watermelon (ClDIR02, 3, and 12) and two proteins from cucumber (CsDIR06 and 09) were classified in this clade. This classification was based on the proteins from dicot plant species, including *L. usitatissium* and *Picea sitchensis.*

#### 3.3.6. DIR-g

The cluster proteins in this subgroup constitutes a species-specific subfamily. We identified two (ClDIR20 and 21), two (CmDIR02 and 12) and one CsDIR07 DIR genes from watermelon, melon and cucumber unclassified in any other identified group. High sequence divergence was reported from proteins of other species and other groups. This cluster is highly divergent among groups, as already reported in other plant species. Similar species-specific classification was performed in studies done in *L. usitatissium* and *Isatis indigotica*.

### 3.4. Systematic Evolutionary Relationship, Motif Analysis and Structural Gene Diversity

#### 3.4.1. Gene Structure Diversity

**Figure 2:**
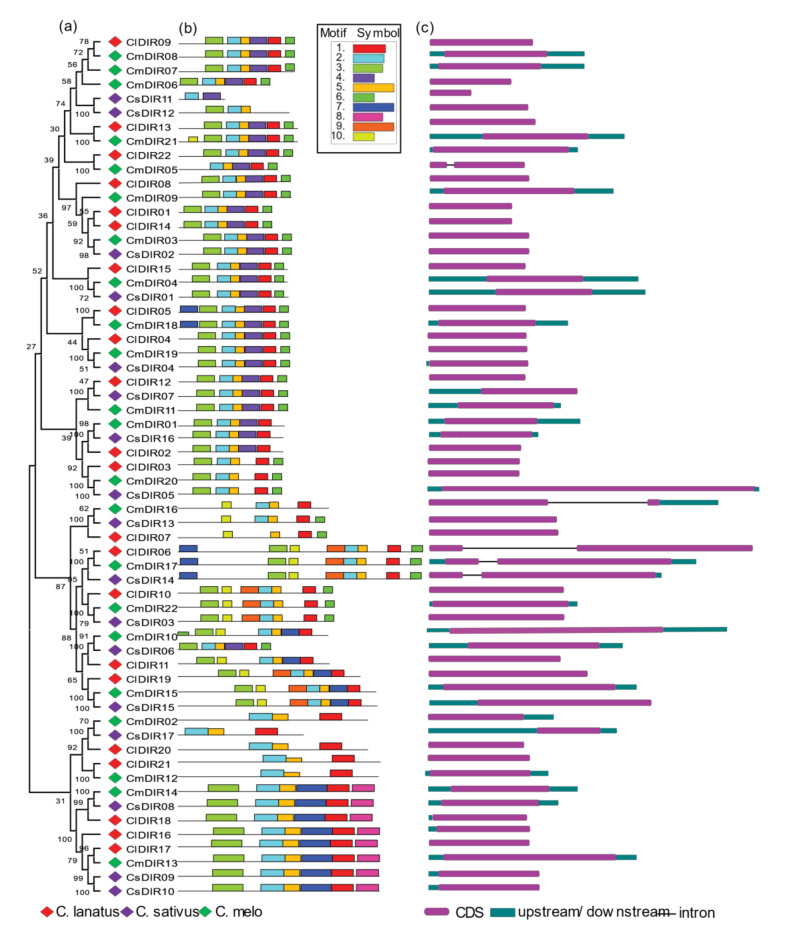
Phylogenetic tree, motif distributions and gene structure of the DIR proteins in different cucurbit species. (**a**) Phylogenetic tree, diamonds in different color represents different species. Red—*Citrullus lanatus*; green—*Cucumis melo*; purple—*Cucumis sativus.* (**b**) Conserved motif analysis of DIR proteins. Motifs were identified using MEME online tool and illustrated in different colors. (**c**) CDS and upstream/downstream structures of DIR genes.

When interpreting phylogenetic relationships within gene families, a structural analysis may provide useful information on duplication of events [40]. Rearrangement or addition of exons and introns may lead to a wide range of gene functions [41]. To examine the structural diversity of DIR genes of major cucurbits species, we presented an unrooted phylogenetic tree with the intron and exon structures of *DIR* genes by comparing with the appropriate corresponding genomic DNA sequences (Figure 2). The number of introns/exons varied in each subgroup and the majority of the members lacked introns, while DIR genes including *ClDIR6*, *CmDIR5/16/17*, *CsDIR14* in *C. lanatus*, *C. melo* and *C. sativus* DIR genes, respectively, possess introns, highlighting the diversity in their structures. Fewer variations in the number of introns lead to the conclusion that they are more conserved over a long period of time. In general, genes with identical intron numbers, locations, and lengths corresponded to closely related siblings found via phylogeny, which supports the validity of the phylogeny classification. Diversification in gene structure confers functional evolution through duplication and subsequent differentiation.

#### 3.4.2. Conserved Motif Distribution

To further evaluate the characteristic regions of DIR proteins, the motif patterns of 61 DIR proteins were analyzed using an online MEME analysis (Figure 2) [42]. Details of the motif are shown in Table 2. Based on the findings of the analysis of the MEME motif, a schematic diagram was designed to characterize the structure of the DIR proteins. In contrast, to the less variability in exon/intron arrangements within subgroups, a similar motif distribution was observed within subgroups. The highly conserved motif-3 was found in all subgroups with few exceptions in subgroup DIR-e and one member of DIR-b/d subgroup. A good distribution of motif-6 was confirmed in all subgroups excluding few members of subgroup-e. Similar motif arrangements were discovered within subgroups with few exceptions including, the motif arrangements in *ClDIR20/21* and *CmDIR12* was observed to be different from other members of this subgroup. *CsDIR11* was discovered with the lowest number of motifs and *ClDIR6*, *CmDIR17* and *CsDIR14* were observed with the highest number of motifs. The DIR-b/d subgroup displayed high diversity in the distribution pattern and number of motifs. In addition, it is worth noting that members of the same clade of the phylogenetic tree showed identical motif organizations with respect to either gene length or motif number with few exceptions.

### 3.5. Prediction of Cis-Element and Gene Ontology (GO) 

#### 3.5.1. Phytohormones and Stress-Responsive *Cis*-Elements in Promoter Region 

**Figure 3:**
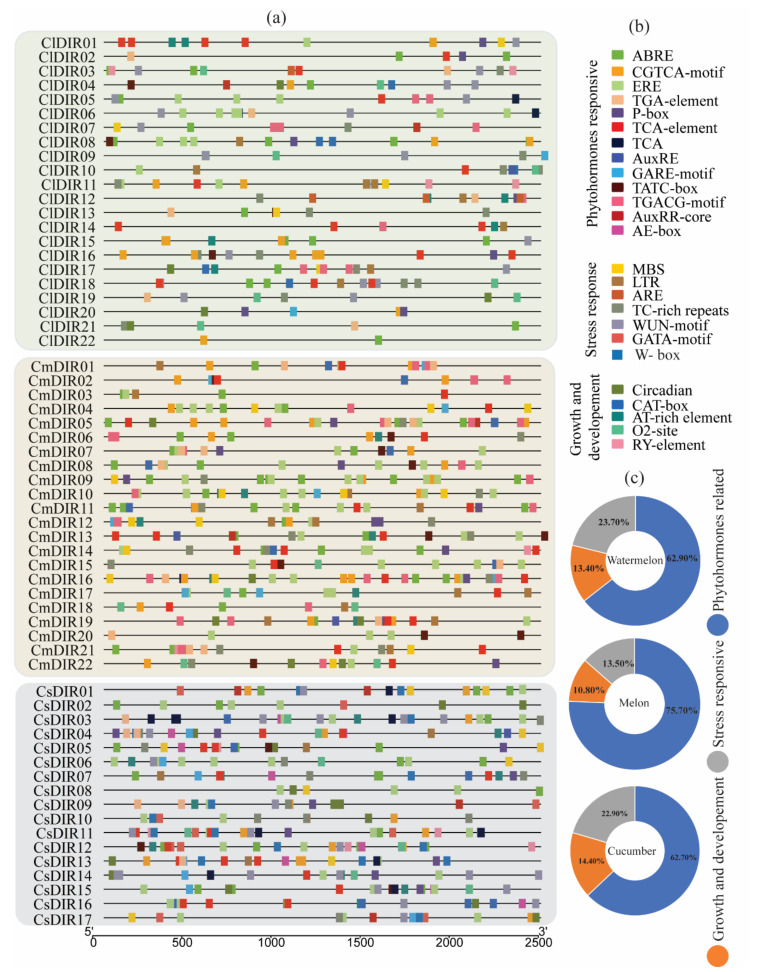
*Cis*-elements in the promoter regions of ClDIR, CmDIR and CsDIR genes. Different colors denote different *cis*-elements and classification is done according to their role in different activities. (**a**) Distribution of important *cis*-elements on promoter region, (**b**) Classification of *cis*-elements according to their function. (**c**) The ratio of various *cis*-elements in three different categories is presented in pie charts.

**Figure 4:**
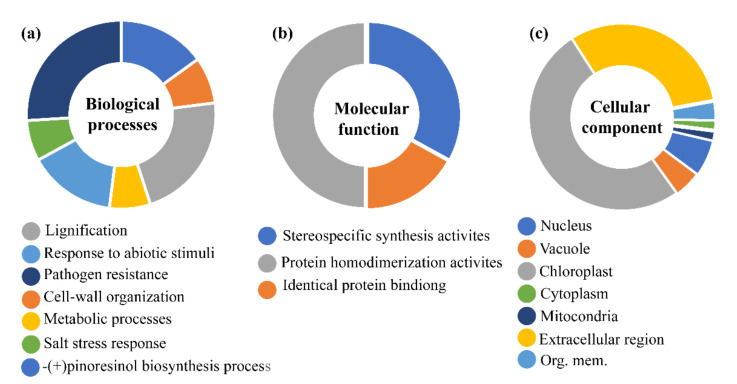
Gene ontology analysis of DIR proteins in different categories. Blast2Go program was utilized to present the biological (**a**), cellular (**b**), and molecular functions (**c**) of different DIR protein in *Citrullus lanatus*, *Cucumis melo* and *Cucumis sativus*. Different colors which are indicated near the pie-charts show functions in different categories.

To gain further insight into the regulatory role of cucurbit DIR genes, in-silico predictions of promoter regions of DIR genes were performed (Figure 3) [43]. A detailed distribution of *cis*-elements among three different species of cucurbits is displayed in Figure 3a. The identified *cis*-elements were classified in three major groups based on their role (Figure 3b). Our result depicted five growth and development related, seven stress response related and 13 phytohormone related *cis*-elements. The growth and development related *cis*-elements included O_2_ cite, CAT-box, Circadian, Ry-element, and At-rich element. The phytohormone related element corresponded to four phytohormones, including salicylic acid (TCA-element), (AuxRR-core, TGA-element) gibberellin (TATC-box, GARE-motif, p-box) and MeJA (TGACG-motif, CGTCA-motif), auxin (AE-box). Stress response related *cis*-elements included low temperature (LTR), wound (WUN-motif), ATR, MBS, TC-rich repeats, and ARE. The detailed analysis of *cis*-regulatory elements showed that 62.70%, 75.70%, and 63% hormone related *cis*-elements were found in watermelon, melon, and cucumber, respectively. A large number of hormone-responsive *cis*-elements were in the promoter region, suggesting that diverse phytohormone induction may regulate their expression. As shown in Figure 4, abscisic acid responsive (ABRE) was identified in the promoters of the majority of ClDIR (except ClDIR6, 9, 10, 11, and 14), whereas gibberellin-responsive *cis*-element (p-box) was identified in five ClDIR genes including *ClDIR01*, *02*, *08*, *12*, *16*, and *20*. In total, 135, 31 and 49 out of 215 *cis*-elements in watermelon were related to hormone, growth development and stress responsive, respectively. Meanwhile, the similar ratio of *cis*-elements in melon and cucumber were observed (Figure 3c). Melon CmDIR genes were identified with the highest number of hormones related *cis*-elements when compared to the other two species. In addition, the highest number of stress responsive and growth and development responsive *cis*-elements were identified in cucumber. Notably, no wound responsive (WUN-motif) *cis*-elements were observed in the promoter sequence of melon DIR genes, whereas 20 and 21 of WUN-motif were reported in cucumber and watermelon. Among all the stress responsive *cis*-elements, WUN-motifs were identified highest in all three species used in our study. Similarly, AT-rich elements in growth and development category were found in the majority of DIR genes. The share of ABRE *cis*-elements was observed highest followed by ERE, TGACG-motif and TCA-element. A wide distribution of stress responsive (TC-rich repeat) *cis*-elements was identified in watermelon (17), melon (16) and cucumber (11). Amongst all, we observed some common *cis*-elements which were directly linked with plant hormone response. Additionally, some significant stress responsive regulatory elements, namely wound and pathogen responsive element (WUN-motif and W-box) and defense and stress-responsive element (TC-rich repeats) were identified in large number. In summary, the presence of important *cis*-elements related to plant stress and phytohormone signifies their involvement in regulating gene expression during different environmental stimuli.

#### 3.5.2. Role in Plant Stress Stimulus and (+)-Pinoresional Biosynthesis Processes. 

To reveal the potential function of DIR genes, we presented a gene ontology (GO) enrichment analysis of the dirigent proteins to predict the putative functions of DIR genes in the biological, molecular and cellular processes (Figure 4) [44]. The prediction of biological processes highlighted potential roles in pathogen resistance, cell-wall origination, lignification, and pinoresinol biosynthesis. Likewise, the prediction in cellular components clarified that most of the genes are involved in chloroplast followed by extracellular regions, which is very similar to our subcellular localization predictions. The present results evidenced the validity of other subcellular prediction methods used in this study. In addition, the prediction of molecular processes suggested that most are involved in protein homodimerization activities followed by stereospecific synthesis and protein-binding activities. The involvement of these proteins in different biological functions such as lignification, pathogens response, cell-wall organization, and response to biotic stress supports the findings of other previous studies. According to GO analysis results in different species of cucurbits, the majority of DIR genes from different species including Arabidopsis (*At*DIRs), rice (*Os*DIRs), flax (*Lu*DIRs), sugarcane (*Sh*DIRs), cotton (*Gh*DIRs), and chili (*Ca*DIRs) showed similar roles, when selected genes from these species were functionally characterized. 

### 3.6. Chromosome Mapping and Synteny Analysis 

#### 3.6.1. Uneven and Wide Distribution of ClDIR, CmDIR and CsDIR Genes on Different Chromosomes

**Figure 5:**
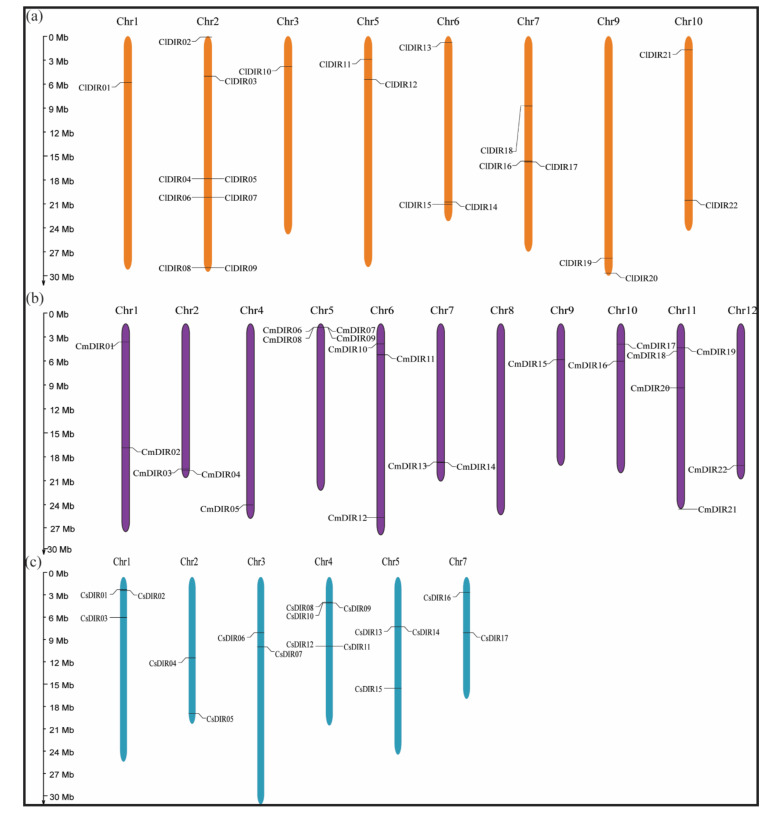
Chromosome mapping of dirigent genes on different cucurbit species. Chromosome numbers are demonstrated on each chromosome and scale is expressed in megabase (Mb). (**a**) Dirigent genes on chromosomes of watermelon (*Citrullus lanatus*). (**b**) Location of dirigent genes on melon (*Cucumis melo*) chromosomes. (**c**) Mapping of dirigent genes on cucumber chromosomes (*Cucumis sativus*).

To study the chromosomal distribution, each DIR from three species of cucurbits was identified and subsequently mapped to a physical position on their respective chromosomes (Figure 5), according to the position and number of chromosomes. All the 61 DIRs were positioned on the Chr1-10, Chr1-12 and Chr1-7 of *C. lanatus*, *C. melo* and *C. sativus* with random distribution. In case of *C. lanatus* no DIR genes were found on Chr11/12 while the maximum number of DIR genes (eight ClDIR) were mapped on Chr2 followed by Chr6 and Chr7 with three ClDIR genes. However only one ClDIR was mapped on Chr1. Majority of the CmDIR genes were located on the tip of chr5. The average of two CmDIR genes were mapped on each chromosome, and only one CmDIR gene was located on short arm Chr9. The chromosome localization of the cucumber DIR genes indicated uneven distribution among Chr1-Chr7. Chr4 carries five (CsDIR8, 9, 10, 11, and 12) CsDIR genes and Chr2, 3 and 7 were observed with only two CsDIR genes on each. Two CsDIR were located on the long arm Chr3 and only two CsDIR were mapped on the short arm of Chr7.

#### 3.6.2. Duplication and Synteny Relationship Analysis

**Figure 6:**
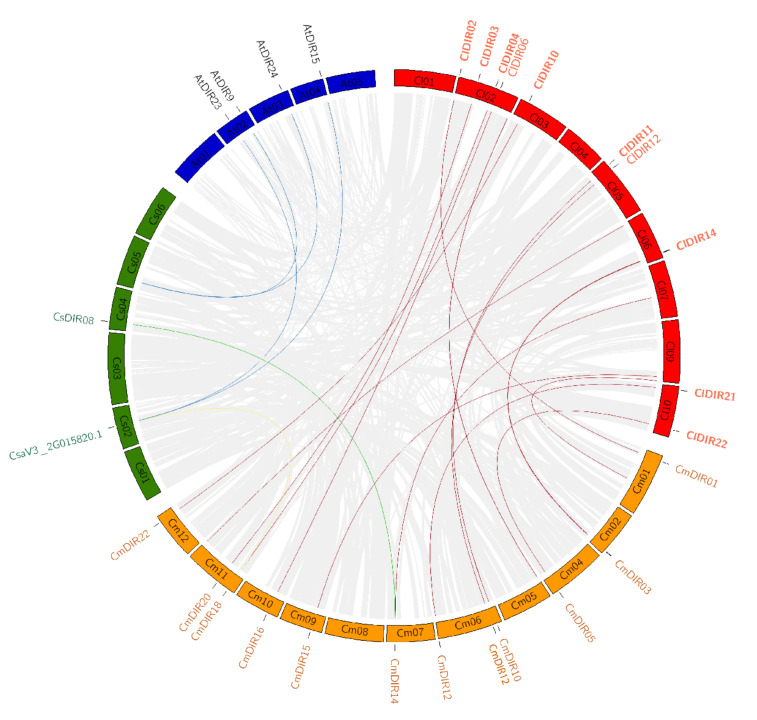
Synteny analysis of dirigent genes of different cucurbitaceous species. In total, 12 DIR genes on nine chromosomes of *C. lanatus*, 11 DIR genes on 11 chromosomes of *C. melo*, 2 DIR genes on 6 chromosomes of *C. sativus*. Background lines of gray color represent all the collinear blocks within different species, while red color is used for *C. lanatus*, green color for *C. sativa*, orange for *C. melo*, and blue shades were used for Arabidopsis. The colored lines from one species to another species depict the orthologous relationships of DIR genes of different species.

To support the presented information about the evolutionary process in cucurbitaceae, we investigated the syntenic relationships of the DIR gene pairs in different species, such as C. *lanatus*, *C. melo*, *C. sativus*, and *A. thaliana*. The synteny analysis revealed that *AtDIR9*, *13*, *15*, and *AtDIR24* have orthologous genes in cucumber on Chr2 and Chr4. Similarly, several orthologous genes were reported in different species of cucurbitaceae. Figure 6 shows a maximum number of orthologous genes between CmDIR and ClDIR. In addition, the analysis with different species of cucurbit genes family revealed that the lowest number of orthologous of DIR genes were present in cucumber and melon. *CmDIR14* melon gene had one orthologous gene *CsDIR8* in cucumber. A high number of orthologous were reported between ClDIR and CmDIR followed by ClDIR and CsDIR and AtDIR and CsDIR. Eight number of duplications were observed for *ClDIR2* followed by four duplications of *ClDIR9*. In terms of species, the genes on Chr2, Chr5, Chr6, Chr9, and Chr10 have duplication of the majority of genes on melon chromosomes. Two genes from cucumber have four orthologous located on Arabidopsis.

### 3.7. Preferential Expression of CsDIR, CmDIR and ClDIR in Root Tissue

Different DIR gene members may exhibit variations in the level of mRNA accumulation among different tissue during the growth and development process of plants. Differential expression in response to stress or sole expression in a certain tissue might indicate a gene function. The tissue-specific expression patterns were evaluated in several plant-tissues such as root, stem, branch, tendrils, leaf, male, female, flower, etc. to explore the potential biological functions of ClDIR, CmDIR, and CsDIR genes during growth and development processes. 

#### 3.7.1. qRT-PCR Expression Analysis of ClDIR Genes in Different Plant Tissues

**Figure 7:**
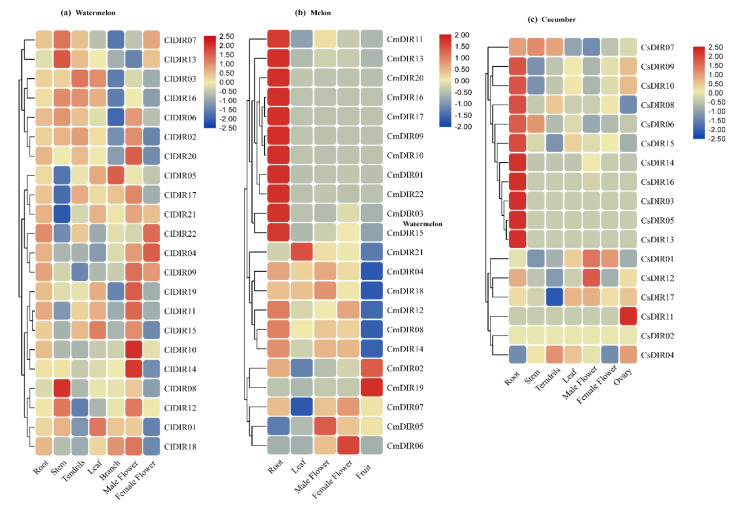
Heat maps representing expression level of all ClDIR, CmDIR and CsDIR genes in different tissues. Normalized Log2 transformation method was used for digital transcript values. (**a**) Heat map representing expression level of ClDIR genes in various tissue (root, tendril, leaf, stem, different sex form flowers, and ovary) based on qRT-PCR expression. (**b**) Expression of CmDIR in various tissues including root, leaf, flower (male, female) and fruit. (**c**) Digital gene expression data of CsDIR genes in various tissues (root, stem, tendrils, leaf, male/female flower, and ovary).

Spatial expression profiling of all the ClDIR genes was performed in the root, stem, branch, tendril, male flower, female flower, and fully opened young leaf (Figure 7a). Based on the heatmap analysis, the ClDIR genes were expressed differentially in various plant tissues. However, the majority of the genes were expressed in root tissues with some exceptions, including *ClDIR8* and *ClDIR13* where no relative transcript level was detected. Moreover, the dominant expression of *ClDIR09*, *10*, *11*, *15*, *17*, and *21* was observed in root tissues in comparison to other tissues analyzed. Similarly, a higher level of expression was seen in leaf tissue for *ClDIR5*, *ClDIR8*, *ClDIR13*, and *ClDIR21*. An involvement of these genes in flowering might be deduced upon their expression in flowering tissue. For instance, many folds transcript abundance was recorded in male flower for *ClDIR09*, *14*, *15*, and *ClDIR20*. Likewise, few genes, including *ClDIR04*, *09* and *ClDIR22* were recorded with the highest expression in the female flower. Two genes (*ClDIR1* and *ClDIR12*) showed significantly elevated expression levels in branch tissue.

#### 3.7.2. Meta-Analysis of Public Expression Data for CsDIR and CmDIR in Various Tissues 

In order to obtain digital transcript expression data for identified DIR genes in cucumber and melon genes, Affymetrix whole-transcriptome data was obtained from the cucurbit genome database (Figure 7). Twelve-week old, Chinese long inbreed line 9930 of cucumber was used to obtain the tissue samples under BioProject-PRJNA312872. In the case of melon, an organ-specific landscape was created by using cv. Charentais, under BioProject- PRJNA383830 (Figure 7b). According to the heatmap created for tissue specific expression, it was concluded that the highest expression of CsDIR and CmDIR was observed in root parts. Some DIR genes were recorded with high expression in different plant tissues. For instance, in cucumber, the expression profile was divided into two subgroups, and the first group consists of the DIR genes with a higher level of expression in root tissue. On the other hand, some CsDIR genes, including *CsDIR11* and *CsDIR12* were expressed in the ovary and male flower, respectively. Furthermore, in the case of melon, three subgroups were obtained. The first group harbored the DIR genes having a higher level of expression. The second group was based on the average level of transcript level in most plant tissues. However, in the third clade *CmDIR19*, *CmDIR5* and *CmDIR6* had the highest transcript level in fruit, male flower, and female flower, respectively. 

### 3.8. Gene Expression Profiling Provides Key Insight into a Potential Role in Disease Resistance

The overview of the available literature showed that dirigent genes are induced by biotic stress in many plant species [20,45]. To explore such responses and to investigate the response of major cucurbit DIR genes after powdery mildew infection, comparative expression profiling was performed by qRT-PCR using unique primers specific to genes (Table S3). Further, digital expression data was retrieved from the melon genome database, and was utilized for heatmap. 

#### 3.8.1. Digital Gene Expression Analysis of CmDIR Genes in Resistant and Sensitive Cultivars in Response to Powdery Mildew Infection

**Figure 8:**
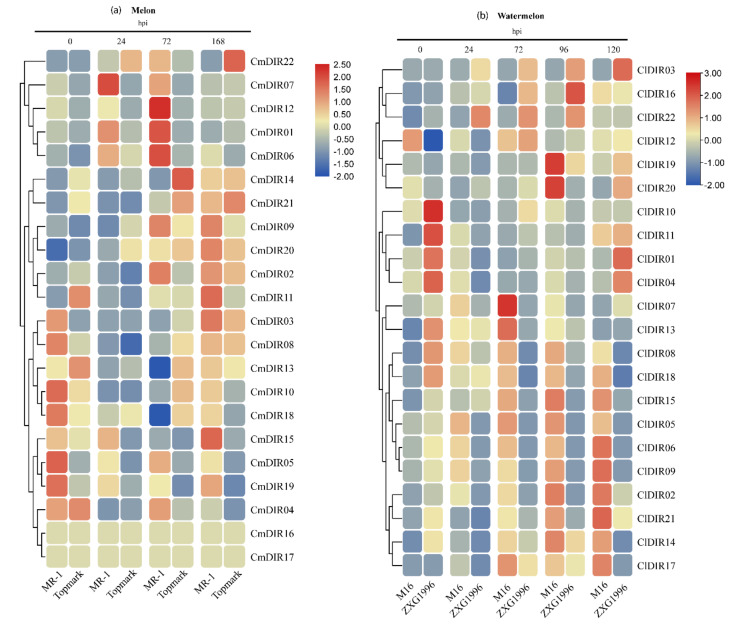
Heatmap of transcription profiling (Log2-based RPKM values (melon) and qRT-PCR (watermelon)) of CmDIR and ClDIR genes in resistant and sensitive cultivars of *C. melo* and *C. lanatus*. (**a**) The heat map representing response of MR-1 (resistant) and Topmark (susceptible) cultivars after different times (hours) post inoculation. (**b**) Heatmap generated from the qRT-PCR data (log2 transformed) obtained from M16 (resistant) and ZXG1996 (susceptible) cultivars of watermelon after PM inoculation.

The expression profile was generated using MR-1 (powdery mildew resistant) and Topmark (powdery mildew susceptible) at different time periods post-inoculation (Figure 8). According to the results, the expression of CmDIR was divided into three clades. The first clade included the genes with strong upregulation after 72 hpi in resistant cultivar. This group consisted of *CmDIR12*, *CmDIR1* and *CmDIR6* genes. The second clade displayed high expression in resistant cultivar at 72 and 168 hpi. Transcript abundance of *CmDIR9*, *CmDIR2*, *CMDIR03*, and *CmDIR11* was recorded higher in resistant cultivar. The DIR members in the third clade showed upregulated expression in resistant cultivars during earlier stages of infection and *CmDIR15* was observed with a higher level of infection in resistant cultivar after 168 hpi. In summary, the CmDIR genes were upregulated in resistant cultivar when compared to sensitive cultivar as well as upregulation was observed in transcript level of CmDIR genes after disease inoculation.

#### 3.8.2. Gene Expression Profiling of Resistant and Sensitive Cultivar against Powdery Mildew Infection 

Watermelon cultivars M16 (powdery mildew resistant) and ZXG1996 (powdery mildew susceptible) were inoculated with powdery mildew race ‘2F’, and the expression levels were analyzed by qRT-PCR at five different time periods (Figure 8b). The results illustrated that the transcript accumulation of majority of ClDIR genes were ubiquitous. In details, *ClDIR2* and *ClDIR6* were expressed only in resistant cultivars. In contrast, *ClDIR03* was expressed only in sensitive cultivar at different time periods after disease inoculation. Five genes (*ClDIR2*, *ClDIR6*, *ClDIR8*, *ClDIR17*, *ClDIR18*) exhibited preferential expression in resistant cultivar. *ClDIR19*, *ClDIR20*, and *ClDIR16* showed an upregulation expression in both the cultivars during all the time periods post-inoculation*. ClDIR5* and *ClDIR8* apparently exhibited abundant expression in resistant cultivar in comparison to susceptible cultivar. Notably, expression of some genes (*ClDIR12* and *ClDIR13*) declined at the later stage of infection in both the cultivars. The expression of *ClDIR5*, *ClDIR6*, *ClDIR8*, and *ClDIR9* was recorded mostly in the resistant cultivar, and the expression gradually increased and attained maximum after 72hpi. The results of the expression profile of ClDIR genes under powdery mildew inoculation suggest their probable involvement in disease resistance mechanism.

## 4. Discussion

Dirigent proteins are essential regulators for plant abiotic and biotic stress tolerance, initially identified for regio- and sterio selective coupling in the process of lignin biosynthesis [3]. Apart from their role in lignin biosynthesis, these proteins are speculated to be involved in various biological processes, including plant biotic stress responses [46]. These proteins are encoded by a multigene family in different vascular plants, with often unknown functions. The dirigent family has been described in a few species. However, there is a paucity of information about the dirigent gene family and gene family members in important cultivated species of the cucurbitaceae family.

### 4.1. Functional Diversification among Subgroups from DIR-a to DIR-f

Dirigent proteins belong to a multigene family and are found in major terrestrial plants [47]. The number of dirigent proteins varies in different plant species. For instance, 26, 19, 35, 29, and 54 dirigent genes have been reported in *A. thaliana* [3,4], *I. indigotica* [48], *Picea glauca* [19], *B. rapa* [17], and *O*. *sativa* [48], respectively. In cucurbitaceae, there are 22 DIR in *C. lanatus*, 22 in *C. melo*, 17 in *C. sativus*, 20 in *C. pepo*, 19 in *C. moschata*, and 23 in *L. siceraria* (Table S1). For further relationships, DIR genes of melon, watermelon and cucumber were utilized. The identified proteins were grouped to six widely known subgroups based on their phylogenetic analysis and classification was carried out according to previous studies in different crops. According to sequence similarity and DIR domain based classification of DIR proteins in Arabidopsis [3,20,49] the ClDIR, CmDIR and CsDIR genes were classified in DIR-a, DIR-b/d and DIR-e. Since there was no classification for DIR-c, DIR-f and DIR-g subgroup in Arabidopsis the ClDIR, CmDIR and CsDIR proteins were classified in this group based on the clustering of orthologs identified in other species. DIR-a subgroup is exclusively classified for its function and the majority of the genes from this subgroup are known for (+)-pinoresinol formation [50]. In total, eight genes from three cucurbits were grouped in this clade. Similarly, other DIR orthologs with established functions including *AtDIR5* and *6* from Arabidopsis, *FxiDIR1* gene from *Forsythia intermedia*, and *ScDIR1* from *Schizandra chinesis* were classified in DIR-a [19,51]. Further, the subgroup DIR-b/d was characterized for stereoselective coupling reaction on the hemigossypol (substrate), to form (+)-gossypol in cotton and its role in pterocarpens biosynthesis. This group comprises the highest number of proteins in this group, suggesting a wider functional divergence with the group. Notably, sometimes group-b and group-d classified in the same cluster, perhaps sequences in these groups have close evaluation history and sequence similarities. Less information is available for DIR-c subgroup and the only known function of this subgroup is related to defense against pathogens [45]. Group-c is considered as specific to monocots and in our results, we demonstrated that none of the identified genes from cucurbits cluster in this category. Some genes from previous studies are demonstrated in group-c. Moreover, DIR-e and DIR-f subfamilies were assumed to be involved in lignin deposition in the Casparian strip [52] and defense against insect and wounding [49], respectively. It was reported in previous studies that a few genes were not in cluster with any known groups, and these kinds of outing are classified as a DIR-g, which is a species-specific group [19]. Five proteins in our study were reported in group-g with unknown functional characters. DIR-c group members showed their highly conserved nature compared to other subfamilies [53,54]. In contrast to the previous hypothesis about the functions of dirigent proteins, that only DIR-a genes are involved in lignin biosynthesis process [4], recent studies showed few members of DIR-b/d subfamilies were also involved in lignin metabolisms, such as *GhDIR1* and *GmDIR22* [54,55]. Overall, distribution patterns of the DIR genes in subgroups were observed similar to the previous studies, the maximum number of DIRs were clustered under DIR-b/d subgroup while least numbers in DIR-a and DIR-f subgroups. The similar arrangements in motif distribution among subfamilies support the evolutionary classification by phylogenetic tree [46,56] 

### 4.2. Gain in Introns during DIR Diversification

The majority of DIR genes lacked introns and few exceptions were observed in selected genes, including *CmDIR05*, *CmDIR16*, *CmDIR17*, *ClDIR06*, and *CsDIR14* possess one intron. Evolution of introns has been associated with alternative splicing events, which leads to functional diversity among encoded proteins [57,58]. The discovery of introns containing in few genes of CmDIR, ClDIR, and CsDIR seems to have the evolutionary linkage between different subgroups. The addition of introns in genes is an advantageous event for expression and regulation [58]. Typical ClDIR, CmDIR, and CsDIR genes exhibited higher similarity in the domain arrangement and diversity in gene structures, implying an evolutionary relationship between genes. It also shows that dirigent genes share a common ancestor and some similarity in biological function [29,56]. 

### 4.3. Wide Distribution of DIR on Different Chromosomes and the Role of Duplication in Gene Evolution

Various mechanisms are involved in the expansion of gene families and gene duplication is an important event that leads to the expansion of various gene families. Gene duplication is considered as a prevailing feature in plant genomes. Large scale segmental duplication or small-scale tandem duplication plays a crucial role in maintaining a high number of gene copies in a gene family. In our synteny analysis, multiple numbers of pairs were detected between CmDIR and ClDIR genes. A large number of orthologous genes indicated the evolution of these genes could be possibly by whole genome segmental duplication events. The large numbers of gene duplications could be possibly facilitated by gene expression, gene organization and plant–pathogen response [59,60]. Moreover, the CmDIR, CsDIR and ClDIR, exhibited several orthologous genes, implying these genes had undergone nonfunctionalization, subfunctionalization, or neofunctionalization [61,62]. The detailed study of flax DIRs showed that neofunctionalization was the major event in the evolutionary expansion of these genes [19].

### 4.4. Transcript Accumulation Patterns in Various Plant Tissues 

The distinctive expression pattern of CmDIR, CsDIR, and ClDIR may provide important clues to predict their physiological functions. The expression of genes in tissue is a common characteristic of the gene specific protein family in plants, which is often related to the function of particular tissues. The public data of transcript expression in melon, cucumber and qRT-PCR expression in watermelon in various tissues of different developmental growth stages, subsequently systematic clustering indicated that DIRs could play multiple roles in growth and development processes. DIR genes were expressed ubiquitously in various plant parts. For instance, the majority of the DIR genes from different cucurbitaceae species were found to be preferentially expressed in roots, which is consistent with the previous study performed on *B*. *rapa* and *I*. *indigotica*, where comparatively higher expression levels were observed in roots [17]. However, in *C. annuum* flower tissues exhibited higher expression [29]. Apart from expression in roots, several genes were highly expressed in some specific plant tissues, such as, in case of watermelon, *ClDIR5*, *8* and *13* in leaves, *ClDIR4*, *9* and *22* in female flower, *ClDIR1* and *12* in branch tissues, suggesting their specific role related to these tissues. In addition, a similar pattern of expression in root was recorded in CmDIR and CsDIR. The higher expression of DIRs genes in floral organs is also consistent with the observations made in *Medica turncatula* and Arabidopsis, where selective genes such as *MtDIR10*, *AtDIR8*, *12* and *20* exhibited comparative higher expression in flowers [3]. *ClDIR14*, *ClDIR10* and *ClDIR20* showed higher expression in male flowers, whereas, *ClDIR04* and *22* had higher expression in female flowers. Similar higher expression was observed for *CsDIR11*, *CsDIR12* and *ClDIR01* which showed higher expression level. The melon genes including *CmDIR06*, *CmDIR05*, and *CmDIR21* exhibited higher expression in different floral organs.

### 4.5. Cultivar Specific Inducible Expression Pattern of ClDIR and CmDIR Response to PM 

PM is a widespread disease of many plant species and is caused by obligate biotrophic fungus [63]. The infection process commences with the landing of spores on the plant surface. The most crucial step in powdery mildew infection is the successful invasion of the first epidermal cell. The plant cell architecture dramatically changes for the accommodation of the haustorial complex and here, the role of lignin monomers is important to mention. Lignification has the potential role to act in several ways in plant defense and it is a well-known to be involved in plant–pathogen interaction [64]. The role of DIRs in modulating the phenylpropanoid pathway, biosynthesis of lignin units and lignans has attracted the research community to uncover the function of these proteins in various stressors. To the date, AtDIR protein family is not fully characterized and only two (*AtDIR5/6*) were found to involve in stereoselective radical-radical coupling [4]. Not much evidence is available to link AtDIR genes with disease response. Nevertheless, DIRs in various pathogen infections, including the infection of anthracnose causing *Colletotrichum gleosporioides* on the *Physcomitrella patens* resulted in increased incorporation of phenolic compounds as well as modifications in cell wall, which was found to be strongly related with the induction of a DIR encoding genes [14]. Similarly, the differential expression in the transcript of *BrDIR12* was recorded after infection of *Fusarium oxysporum* in *Brassica oleracea* [17]. *TaJAl* with dirigent domain in barley were found to contribute in enhancing disease resistance against PM [18]. According to our expression assessment outcomes, the expression level of DIRs in plant tissues during the disease developmental stage reflects their feasible direct or indirect active participation in different stages of infection. As inferred from the qRT-PCR data, in case of watermelon, *ClDIR02*, *5* and *8* exhibited constitutive higher expression in resistant cultivars, which is much similar to the finding of Elena Sánchez-Elord et al., in which the higher transcript level was observed in resistant cultivars [65]. *SofDIR16* gene from sugarcane was highly expressed in resistant cultivar after inoculation of *Sporisorium scitamineum* [65]. On the other hand, the similar patterns were observed from melon heat map analysis, certain DIR genes, including *CmDIR01*, *6*, *7*, *12*, and *15* were highly upregulated in resistant cultivar. As per the available literature to date, elevated expression of DIR genes in different crops against biotic stress was observed, perhaps suggesting their possible role in disease response [66,67,68,69]. Here, our *cis*-element analysis revealed that DIR genes in melon, cucumber and watermelon harbor a large number of phytohormone and stress responsive *cis*-elements. The detailed study of *AtDIR5* showed a strong induction in expression level against methyl jasmonate (MeJA). This finding also relates its role in resistance mechanism because MeJa is an important signal mediator in response to stress. Moreover, our results corroborate the former studies related to the *cis*-element in promoter regions, elements related to stress and defense related hormones were found in upstream sequences. Element responsive to salicylic acid and MeJA have been identified in many ClDIR, CmDIR and CsDIR genes, indicating that perhaps these genes have a functional role associated with phytohormones. The role of these genes against fungus disease is studied in some crops. The inoculation of *P. sativum* root with fusarium enhanced their enhanced expression level leading to pinoresinol accumulation in roots. Similarly, some genes including *LuPLR1* showed higher expression when flax cell suspension was exposed against *Fusarium oxiysporum* [70,71].

DIR genes were detected in both resistant and susceptible cultivars in response to PM inoculation. Expression of *ClDIR5* and *ClDIR8* was found to be higher in leaf tissues of watermelon and in a constitutive way the expression was recorded much higher in resistant cultivars. With the increase of inoculation time, the gradual increase in expression was observed in resistant cultivars, and this phenomenon may be due to the involvement of these genes in post-inoculation disease responses. A sharp rise in expression of *ClDIR05*, *6*, *8*, and *9* was recorded in resistant cultivars depending on the inoculation time, i.e., the gradual increase in expression level was observed as post-inoculation time increased. This is most likely due to pathogenesis leading to a local rise in expression [72]. These comparative results provide support to the hypothesis that DIR genes are primarily related to plant-pathogen infection. The higher level of transcript in later stages of infection supports our hypothesis that it has an important role in later stages of disease development. 

## 5. Conclusions

We identified 22 DIR genes in watermelon, 22 in melon and 17 in cucumber, distributed unevenly on different chromosomes. Phylogenetic analysis revealed the existence of six subgroups based on domain and structural characteristics. Furthermore, our findings provided useful information for the evolution and functional divergence of DIR genes in plants. In addition, our research provides comprehensive information about the involvement of these genes in activities related to phytohormones, stress response and lignin biosynthesis based on *cis*-element and GO analysis, respectively. The dominant expression of these genes in root tissue provided evidence for the possible involvement of these genes in root related functions. RNA-seq and qRT-PCR analyses indicated that ClDIR and CmDIR could be defense-responsive genes in different species of cucurbits, when challenged by powdery mildew stimuli, while some genes were explicitly upregulated in resistant cultivars in both crops. Here the combination of in silico and qRT-PCR analysis provided useful information for functional studies in biotic stress. Based on the available indications, it seems that DIR genes are critically involved in plant defense, probably by their role in secondary cell wall metabolism and production of defense related compounds. This study lays the foundation for gene functional validation studies in cucurbitaceous vegetable crops to identify the involvement of DIRs in lignin polymerization and possible involvement in plant–pathogen interaction. 

## Figures and Tables

**Table 1:** List of identified dirigent proteins in major cucurbits, sequence characteristics and protein properties. MW—molecular weight; pI—isoelectric point; chlo—chloroplast; extr—extracellular space; mito— mitochondria; vacu—vacoule; nucl—nucleus.

Plant Species	Gene ID	CDS (bp)	No of AA	MW (kDa)	PI	Instability Index	Aliphatic Index	GRAVY	Subcellular Localization
***C. lanatus***	ClDIR01	474	157	17.273	7.10	27.66	83.25	−0.067	chlo
	ClDIR02	525	174	18.864	6.55	24.12	98.62	0.329	extr. sp
	ClDIR03	525	174	18.441	9.64	9.42	99.77	0.272	extr. sp
	ClDIR04	564	187	20.495	8.59	30.7	88.61	−0.079	extr
	ClDIR05	555	184	19.795	9.19	30.59	92.66	0.147	extr
	ClDIR06	1191	396	41.460	4.36	43.18	79.34	−0.14	extr
	ClDIR07	738	245	27.495	9.63	33.34	68.86	−0.329	extr
	ClDIR08	567	188	20.582	10.04	28.82	100.64	0.176	extr
	ClDIR09	588	195	21.721	9.85	31.9	83.54	−0.05	chlo
	ClDIR10	768	255	27.451	5.33	44.6	77.22	−0.292	chlo
	ClDIR11	753	250	25.619	5	38.14	88.6	0.221	extr:
	ClDIR12	549	182	20.060	5.41	38.82	93.63	0.116	vacu
	ClDIR13	603	200	22.527	6.51	42.14	89.8	−0.007	chlo
	ClDIR14	474	157	17.482	6.58	44.91	75.1	−0.207	extr
	ClDIR15	552	183	20.038	7.89	36.89	92.19	0.236	extr
	ClDIR16	579	192	21.159	7.74	44.41	86.35	0.169	extr
	ClDIR17	573	190	21.150	8.38	22.75	83.74	0.127	extr
	ClDIR18	558	185	20.586	8.95	32.29	84.43	0.039	chlo
	ClDIR19	906	301	30.69	4.92	35.85	77.74	−0.052	nucl
	ClDIR20	546	181	20.235	9.69	38.17	85.75	−0.223	chlo
	ClDIR21	582	193	21.146	7.34	27.1	94.87	0.172	extr
	ClDIR22	579	191	20.730	9.74	52.29	93.93	0.231	org. mem.
***C. melo***	CmDIR01	531	176	19.135	7.78	28.23	93.69	0.207	chlo
	CmDIR02	546	191	20.924	9.01	24.49	92.77	0.138	vacu
	CmDIR03	573	190	20.663	9.12	38.02	81.05	−0.091	extr
	CmDIR04	552	183	19.983	6.06	34.77	93.17	0.245	chlo
	CmDIR05	501	166	17.805	9.3	48.42	76.93	0.172	chlo
	CmDIR06	465	154	17.041	10.04	29.77	74.16	−0.212	extr
	CmDIR07	588	195	21.555	9.48	34.81	85.54	0.004	chlo
	CmDIR08	588	195	21.646	9.52	29.96	84.51	−0.007	chlo
	CmDIR09	570	189	21.189	9.49	28.92	93.39	0.105	nucl
	CmDIR10	747	248	25.332	5.16	31.55	90.48	0.234	extr
	CmDIR11	549	182	19.864	4.99	33.04	89.34	0.149	chlo
	CmDIR12	576	191	20.924	9.01	24.49	92.77	0.138	nucl
	CmDIR13	579	192	21.353	7.72	26.75	86.41	0.189	chlo
	CmDIR14	564	187	20.963	8.48	40.91	76.15	0.01	extr
	CmDIR15	984	327	33.789	5.08	37.1	82.57	−0.003	extr
	CmDIR16	750	244	27.605	9.67	42.98	65.94	−0.396	chlo
	CmDIR17	1185	394	41.230	4.29	47.64	75.33	−0.166	chlo
	CmDIR18	555	184	19.947	9.16	30.57	86.9	0.055	chlo
	CmDIR19	564	187	20.493	9.32	39.66	89.63	−0.028	chlo
	CmDIR20	519	172	18.21	9.57	12.26	95.29	0.197	chlo
	CmDIR21	603	200	22.350	5.94	38	100	0.084	chlo
	CmDIR22	777	258	27.890	5.96	41.65	76.32	−0.288	chlo
***C. sativus***	CsDIR01	552	183	19.943	6.06	32.24	90	0.255	nucl
	CsDIR02	573	190	20.685	9.39	45.56	85.16	−0.055	chlo
	CsDIR03	774	257	27.810	5.69	46.08	75.53	−0.284	chlo
	CsDIR04	564	187	20.450	8.93	36.84	88.07	−0.004	chlo
	CsDIR05	519	172	18.340	9.75	10.99	91.28	0.119	cyto
	CsDIR06	750	249	25.447	5.35	32.07	90.88	0.248	vacu
	CsDIR07	549	182	20.146	5.14	26.75	86.1	0.056	chlo
	CsDIR08	561	186	20.951	8.6	30.79	87.04	0.111	chlo
	CsDIR09	576	191	21.309	8.86	28.57	87.8	0.169	chlo
	CsDIR10	576	191	21.309	8.86	28.57	87.8	0.169	chlo
	CsDIR11	237	78	8.696	9.85	36.49	70	−0.338	chlo
	CsDIR12	561	186	21.484	9.62	31.97	80.22	−0.294	ogr. mem
	CsDIR13	729	242	27.432	9.67	40.72	64.05	−0.413	chlo
	CsDIR14	1185	394	41.42	4.31	47.97	77.54	−0.177	chlo
	CsDIR15	990	329	34.014	5.08	33.9	79.12	−0.011	chlo
	CsDIR16	525	174	18.969	5.4	28.27	97.53	0.274	extr
	CsDIR17	363	120	13.329	6.91	47.2	73.08	−0.203	mito

**Table 2:** Identified motif of ClDIR, CmDIR and CsDIR genes. Logo chart of the conserved motifs, sequence, site, width and E values were obtained from MEME (Multiple Em for Motif Elicitation).

Motif	E Value	Site	Width	Consensus Sequence	Motif Logo
1	5.8e-629	59	23	RELPVVGGTGKFRFARGYATAKT	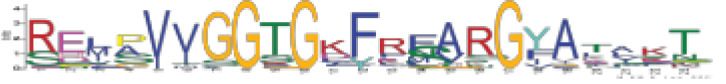
2	2.6e-524	60	22	FGTVFVIDBPLTEGPELGSKLV	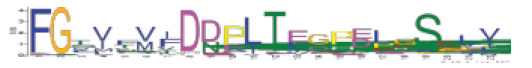
3	7.6e-417	51	21	TNLVFYMHDILSGKNPTAIAV	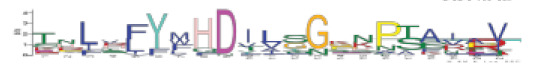
4	2.4e-328	60	15	GRAQGFYVSSSQDGF	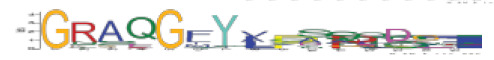
5	2.6e-357	30	29	LLMAMNFAFTSGKYNGSSJTILGRNPVME	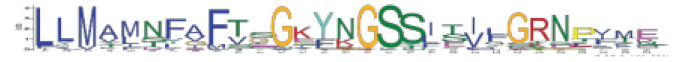
6	2.9e-187	40	15	DTGDAVVEYNVYVLH	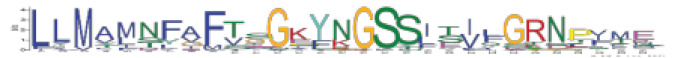
7	2.0e-124	19	29	WLAFSFMFNSTEYKGSJNFVGADPJMVKT	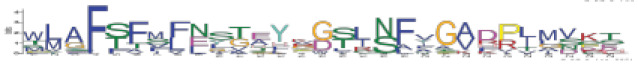
8	1.9e-098	8	21	DAFEGEVYFRLRVDIKFYECW	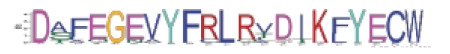
9	1.9e-080	9	29	GGLNGNEDNNQPFVTAGQLPSGVTLQQLM	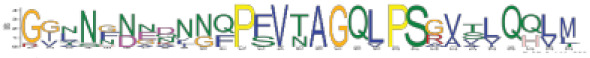
10	7.5e-066	20	15	SSQLPFSKPNKFFPP	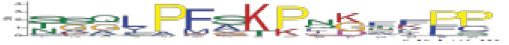

## Supplementary Materials

The following are available online at https://www.mdpi.com/2073-4425/12/3/326/s1, Table S1: Genomic information and identification of dirigent genes in major cucurbitaceous crops, Table S2: Genomic information of the sequences used for phylogenetic tree, Table S3: Specific primers of ClDIR genes used for qRT-PCR in this study.
