# Supplementary material for: Comparative Analysis, Characterization and Evolutionary Study of Dirigent Gene Family in Cucurbitaceae and Expression of Novel Dirigent Peptide against Powdery Mildew Stress
Source: Genes (Basel). 2021 Feb 24;12(3):326. doi: 10.3390/genes12030326 (PMC7996225; doi:10.3390/genes12030326)
Supplement: Supplementary file 1 [file genes-12-00326-s001.pdf]

Supplementary Table S1. Genomic information and identification of dirigent genes in major cucurbitaceous crops. (*Citrullus lanatus, Cucumis melo, Cucumis sativus, Cucurbita pepo, Cucurbita moschata and Lagenaria siceraria*).

Species name	No. of genes	Number of identified genes, Gene Bank accession number	Database/Source
C. lanatus	**22**	Cla003948, Cla007659, Cla010454, Cla020305, Cla020304, Cla020112, Cla020111, Cla003180, Cla003179, Cla007028, Cla019346, Cla021456, Cla021796, Cla019000, Cla005493, Cla001392, Cla001396, Cla000760, Cla004709, Cla019036 Cla008949, Cla017564	[38]
C. melo	**22**	MELO3C018823, MELO3C012606, MELO3C017279, MELO3C017251, MELO3C009601, MELO3C014668, MELO3C014667, MELO3C014666, MELO3C014665, MELO3C006347, MELO3C016325, MELO3C023825, MELO3C006716, MELO3C013719, MELO3C023824, MELO3C002419, MELO3C016328, MELO3C025485, MELO3C020795, MELO3C019288, MELO3C020796, MELO3C022433	[38]
C. sativus	**17**	Csa1G015640, Csa1G015830, Csa1G058130, Csa2G264050, Csa2G416780, Csa3G133310, Csa3G166330, Csa4G050220, Csa4G050240, Csa5G179750, Csa4G050250, Csa4G280610, Csa4G280620, Csa5G179740, Csa5G505160, Csa7G043060, Csa7G238990	[38]
C. pepo	**20**	Cp4.1LG01g04560, Cp4.1LG01g00150, Cp4.1LG01g10610, Cp4.1LG01g10620, Cp4.1LG03g00040, Cp4.1LG03g18290, Cp4.1LG05g05240, Cp4.1LG05g13180, Cp4.1LG05g13120, Cp4.1LG05g13130, Cp4.1LG08g05350, Cp4.1LG08g03040, Cp4.1LG09g09110, Cp4.1LG13g09560, Cp4.1LG14g02380, Cp4.1LG14g02290, Cp4.1LG15g07390, Cp4.1LG16g04310, Cp4.1LG16g09090, Cp4.1LG18g07240	[38]
C. moschata	**19**	CmoCh02G003170, CmoCh02G003180, CmoCh02G012440, CmoCh04G002460, CmoCh04G012860, CmoCh04G012870, CmoCh04G027380, CmoCh06G012820, CmoCh10G002740, CmoCh10G003000, CmoCh14G004020, CmoCh14G019100, CmoCh15G004330, CmoCh16G006220, CmoCh16G013330, CmoCh19G011360, CmoCh20G000440, CmoCh20G000450, CmoCh20G006340	[38]
L. siceraria	**23**	Lsi02G005580.1, Lsi02G016820.1, Lsi03G017580.1, Lsi04G008520.1, Lsi04G008530.1, Lsi05G015960.1, Lsi05G017990.1, Lsi06G013580.1, Lsi06G013920.1, Lsi07G009150.1, Lsi07G009170.1, Lsi07G009180.1, Lsi07G010490.1, Lsi07G014300.1, Lsi09G018340.1, Lsi10G001150.1, Lsi10G004700.1, Lsi10G004720.1, Lsi10G009810.1, Lsi10G009820.1, Lsi10G009830.1, Lsi10G009840.1, Lsi11G014430.1	[38]

Supplementary Table S2. Genomic information of the sequences used for phylogenetic tree.

Species	Name	Accession No
Arabidopsis thaliana	AtDIR1	At5g42510
	AtDIR2	At5g42500
	AtDIR3	At5g49040
	AtDIR4	At2g21110
	AtDIR5	At1g64160
	AtDIR6	At4g23690
	AtDIR7	At3g13650
	AtDIR8	At3g13662
	AtDIR9	At2g39430
	AtDIR10	At2g28670
	AtDIR11	At1g22900
	AtDIR12	At4g11180
	AtDIR13	At4g11190
	AtDIR14	At4g11210
	AtDIR15	At4g38700
	AtDIR16	At3g24020
	AtDIR17	At3g58090
	AtDIR18	At4g13580
	AtDIR19	At1g58170
	AtDIR20	At1g55210
	AtDIR21	At1g65870
	AtDIR22	At3g13660
	AtDIR23	At2g21100
	AtDIR24	At3g55230
	AtDIR25	At1g07730
Forsythia intermedia	FiDIR1	AAF25357
Gossypium bardadense	GbDIR1	AAS73001
	GbDIR2	AAY44415
Hordeum vulgare	HvDIR1	AAA87042
Oryza sativa	OsDIR8	BAB89617
Picea sitchensis	PsDIR23	ABR27719
	PsDIR28	ABR27724
Schisandra chinensis	ScDIR1	ADR30610
Sesamum indicum	SiDIR1	AAT11124
Tamarix androssowii	TanDIR1	ABE73781
Triticum aestivum	TaDIR1	AAC49284
Tsuga heterophylla	ThDIR1	AAF25367
Zea mays	ZmDIR1	AAF71261
LuDIR33		
LuDIR34		
LuDIR35		
LuDIR31		
LuDIR32		

Supplementary Table S3. **Specific primers of ClDIR genes used for qRT-PCR in this study**

Gene ID	Forward primer (5'-3')	Reverse primer (5'-3')	Note
ClDIR01	CGCTACTGTGTTCGGTGAAGTCAA	GACGGCGTCGGAGATAGCATTC	qRT-PCR
ClDIR02	GCCTTGCTGCCTCCATTTCCAT	CGGTTATCGCATCGTCGGTCAC	qRT-PCR
ClDIR03	TGATTCTCACAACAGCCGCCATT	GCCAGCGTCAGAACCACCAAT	qRT-PCR
ClDIR04	ATCCATCCGCCAGAGTAG	AGTCGCCGATTGGTCGCCTAT	qRT-PCR
ClDIR05	TGTAGCATTCTCCTTCATCC	GCAACTCACGAACGGTAT	qRT-PCR
ClDIR06	ATCCATCCGCCAGAGTAG	GAGGTTGTTGTTGTTGTTGA	qRT-PCR
ClDIR07	TCACCTACCACCAAAGCAACAACAA	AGTCCCAAACAACTCCTCATCAACC	qRT-PCR
ClDIR08	TACGCTTCGACCGCACTGGAT	ACTACCACCGATGACCGACAACT	qRT-PCR
ClDIR09	CAGCCACTTCCGTCTCTA	GTATTCAACAACCGCATCC	qRT-PCR
ClDIR10	GCCATACCGTAGCATTGACCATCAT	ACCGCAAACCCACCAGCATTC	qRT-PCR
ClDIR11	TGGTGCTCTCCCTACTGTGAATGG	CAACTCTGGTGCGGTGGTCAAG	qRT-PCR
ClDIR12	AACTTCCTCCTCCTCTCCATCCTC	TACAGCCCTTTCCCGTCCGATT	qRT-PCR
ClDIR13	TCCAGTCAGGATTTGGGTACATTCG	CCACTTCCTCCAATCACAGACAACT	qRT-PCR
ClDIR14	GCCACTGTGTTCGGTGAAGTCAATA	TTCCTCCGACGACTGGCATCTC	qRT-PCR
ClDIR15	AATGTCGTCGCAGCAGAATGAGTT	CGTTGTAGCCAATCACAGCATCCA	qRT-PCR
ClDIR16	TTCCTCCTCCTCAACCTCACCATT	CCAACCAGGCAGCGAAGATGTT	qRT-PCR
ClDIR17	CCATTGTTGCAGCTCCAGAAGGT	GGTCAGCTCCGACGAAGTTGATG	qRT-PCR
ClDIR18	TCTCTTCTTCCTCCTCCT	TGTTGTCCAGTGTGATTG	qRT-PCR
ClDIR19	GACTTCTTGACGAGCAGCCACAA	GGGATTTGAACCGCCGAGGATG	qRT-PCR
ClDIR20	GCAGACCTGTCCCTCTACATCCAA	TGAACGACGAACGAGCAAACTGT	qRT-PCR
ClDIR21	CACCATCATCACCACCACCATCAC	TGCAAGCCGTCGAAGCCAGA	qRT-PCR
ClDIR22	GCGGTGGTGATGACGGATGATC	TCTCTCTGACGGCGGACATGAC	qRT-PCR
Actin	CCATGTATGTTGCCATCCAG	GGATAGCATGGGGTAGAGCA	
